# Designed NiMoC@C and NiFeMo_2_C@C core-shell nanoparticles for oxygen evolution in alkaline media

**DOI:** 10.3389/fchem.2023.1162675

**Published:** 2023-04-26

**Authors:** Xiang Li, Cristina Giordano

**Affiliations:** Department of Chemistry, Queen Mary University of London, London, United Kingdom

**Keywords:** OER, ternary transition metal, nanoparticles, core-shell structure, graphitic carbon, binary transition metal, metal-metal carbide hybrid, urea glass route

## Abstract

Electrochemical water splitting is one of the most promising and clean ways to produce hydrogen as a fuel. Herein, we present a facile and versatile strategy for synthesizing non-precious transition binary and ternary metal-based catalysts encapsulated in a graphitic carbon shell. NiMoC@C and NiFeMo_2_C@C were prepared *via* a simple sol-gel based method for application in the Oxygen Evolution Reaction (OER). The conductive carbon layer surrounding the metals was introduced to improve electron transport throughout the catalyst structure. This multifunctional structure showed synergistic effects, possess a larger number of active sites and enhanced electrochemical durability. Structural analysis indicated that the metallic phases were encapsulated in the graphitic shell. Experimental results demonstrated that the optimal core-shell material NiFeMo_2_C@C exhibited the best catalytic performance for the OER in 0.5 M KOH, reaching a current density of 10 mA cm^-2^ at low overpotential of 292 mV for the OER, superior to the benchmark IrO_2_ nanoparticles. The good performances and stability of these OER electrocatalysts, alongside an easily scalable procedure makes these systems ideal for industrial purposes.

## 1 Introduction

**SCHEME 1:**
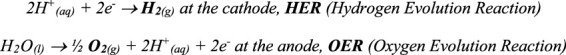
The two semi-reactions in the electrolysis of water.

The electrolysis of water, also known as “water splitting” (Scheme 1) is one of the most promising ways to generate clean and sustainable hydrogen-based energy and great efforts have been made to optimize H_2_ production by the corresponding Hydrogen Evolution Reaction (HER) half-reaction. However, one of the main problems restricting the overall process is the high energy barrier of the other half-reaction, namely, the Oxygen Evolution Reaction (OER).

To ensure an efficient water conversion, the overall process requires the use of suitable electrocatalysts (Pt for HER, and IrO_x_ or RuO_x_ for the OER are current benchmarks, due to their fast reaction kinetics and stability). Yet, the high cost and scarcity of the existing electrocatalysts cannot make their use a long lasting solution. In this respect, multiple classes of materials have been explored, including metal alloys (Jeong et al., 2019; An et al., 2022), oxides (Chen et al., 2022; Eum et al., 2022), hydroxides (Ni et al., 2017; Indra et al., 2022), phosphide (Lin et al., 2019), sulfide (Yan et al., 2021; Shaikh et al., 2022), selenide (Shaikh et al., 2022; Singh et al., 2022), nitrides (Defilippi et al., 2019) and carbides (Niether et al., 2018; Qin et al., 2018; Li et al., 2021) based on non-noble transition metals, i.e., Ni, Mo, Co, Fe and Mn (Kar et al., 2022).

Among these metals, Ni is considered an ideal choice thanks to its abundance, lower price, good conductivity and expected good activity towards the OER. Pure Ni, however, has a high overpotential, and can be (partially) oxidized during the electrochemical cycling, gradually losing its electrocatalytic activity and conductivity.

To improve its stability during electrocatalysis, the introduction of a second metal into the Ni structure has been considered. Beside stabilizing the final structure, the incorporation of a second metal can also enhance conductivity and, thanks to synergistic effects, improve activity. Binary nickel compounds including NiMo, NiFe, NiCu and NiCo (Jeong et al., 2019; Ahsan et al., 2020; Han et al., 2021; Li et al., 2022) have been tested for the OER. NiMo compounds show a promising activity, with a low overpotential of 190 mV at 10 mA cm^-2^ for the OER in alkaline media (Zu et al., 2019), making it one of the best performing catalysts that have been reported so far. In addition to an improved catalytic activity, the corrosion resistance of NiFeMo_2_C@C during the OER process was also enhanced. Nonetheless, despite the promising OER performance of bimetallic NiMo, currently no transition metal based catalysts can fully satisfy the requirements for large-scale commercial water splitting. The challenge to design electrocatalysts with high OER activity as well as long-term stability based on sustainable materials for industrial still remains.

To overcome the challenge, ternary catalysts were recently explored by introducing a third transition metal into the NiMo-based structure. According to theoretical studies, the synergistic effect resulting from the combination of the transition metals in the ternary catalysts should be able to modulate the electronic structures (Luo and Koper, 2022). By incorporating metal elements, the fnal electromic structure and catalytic performance can be turned (Medford et al., 2015; Zhang B and Voznyy, 2016; Ahsan et al., 2020). Therefore, ternary metal catalysts normally show superior electrocatalytic activity and stability compared to bimetallic materials. However, despite the enhanced performance, the synthesis of most ternary catalysts needs to go through a relatively complex synthetic routes, e.g., phosphorylation (Baek et al., 2019) and electrodeposition (Badrnezhad et al., 2021; Zhang P. et al., 2021), which would inevitably limit their use for large-scale applications. Thus, exploring simpler and safer, as well as more efficient ways to modify NiMo-based ternary catalysts remains a challenge.

In general, one of the challenges for the application of transition metal based nanoparticles in the OER process is the lack of long-term stability, mainly due to the agglomeration and corrosion of electrocatalysts during the OER process. A promising approach to overcome these issues is to armour the active core with a functional shell. Thus, core-shell structure of transition metals encapsulated within graphitic carbon shell could guarantee a high efficiency and a long-time operation (Tu et al., 2018). Graphitic carbon shell can improve activity, considering that the majority of the electroactive sites are located on the shells interface (Khani et al., 2019), and can also act as proton transport mediator, while the inner core metal acts as a conductive electron pathway (Baek et al., 2019), which overall enhances the conductivity of the whole system in the OER process. Controlling the composition of transition metal core can also alter the electronic properties of the carbon shells thanks to the core metals induced electronic effects, which enable to turn the binding energies of the OER intermediates and enhance the catalytic activities (Chen et al., 2015; Cui et al., 2016; Yang et al., 2016; Zhang et al., 2017; Tu et al., 2018). The interactions between the metals in the core can also induce interatomic electron transfer, providing additional active catalytic sites (Sanad et al., 2021). The beneficial effect lead by the presence of the carbon phase was also nicely showed by Ahsan (Ahsan et al., 2020), who observed an improved oxidation resistance and a reduced agglomeration of the nanoparticles, alongside a better conductivity. However, few studies have been focusing on the structure of graphitic shell with ternary transition metals as a core.

In this paper, we presented a versatile and straightforward way to incorporate metals into Ni catalyst to prepare binary and ternary metal-based core-shell nanoparticles. Binary NiMoC@C and ternary NiFeMo_2_C@C core-shell nanoparticles were explored and optimised from both morphological and electronic structure point of view, to increase the density of active sites and optimizing the electron structure. For a better understanding of the mechanism, and the role of the metal centers, also the monometallic systems were tested for the OER, showing a much higher overpotential in alkaline media. The graphitic shell provided them with improved catalytic activity and stability comparing to the monometallic systems. Trimetallic NiFeMo_2_C@C showed an even lower overpotential (292 mV at 10 mA cm^-2^) in alkaline media due to synergistic effect, the formation of defects and extra active sites exposed in the graphitic shell.

## 2 Experimental procedure

### 2.1 Synthesis of nanomaterials

**FIGURE 1:**
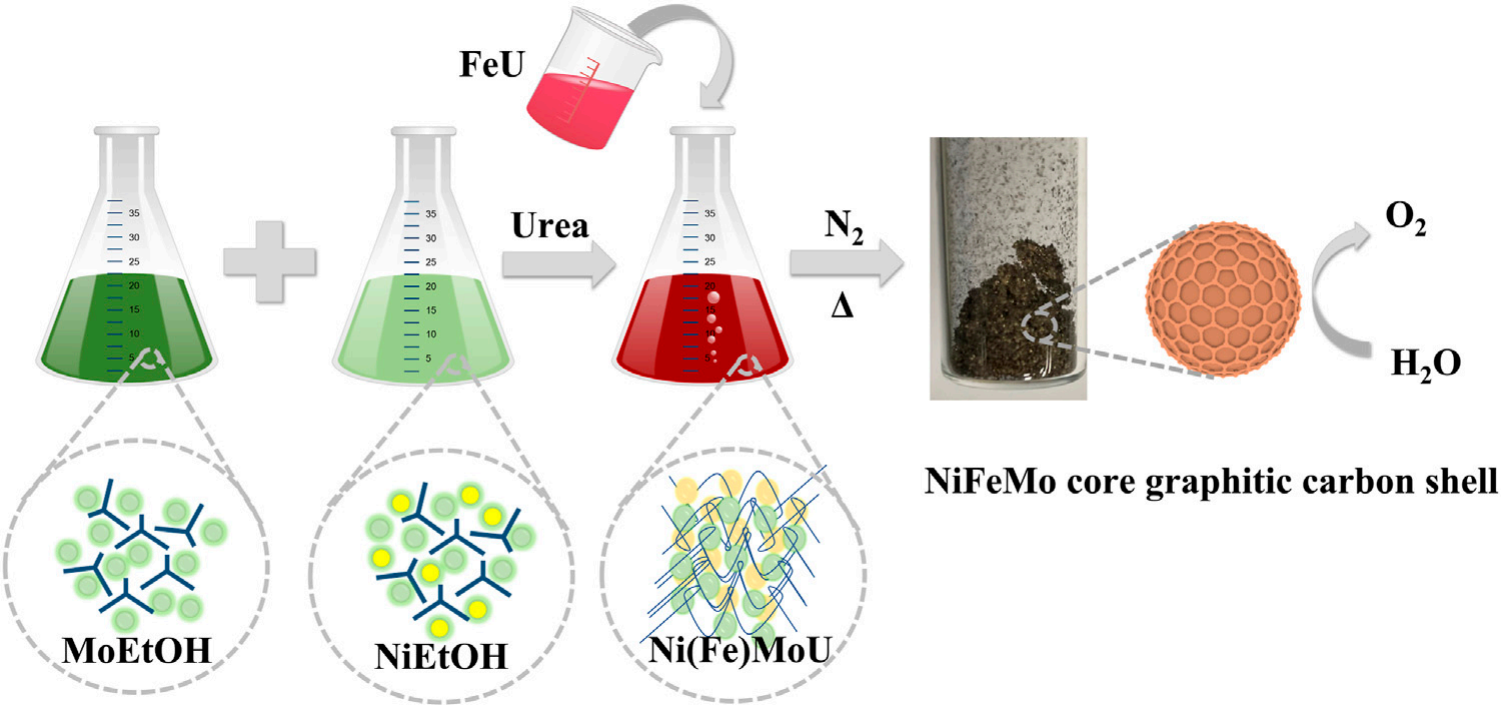
Schematic representation of the urea-glass route.

All chemicals were purchased from Sigma-Aldrich and kept in a glove box filled with N_2_. The synthesis procedure employed is called urea-glass-route (Giordano et al., 2008; Giordano et al., 2009). A schematic representation of the nanoparticles’ synthesis is reported in Figure 1. A suitable amount of Ni and Mo metal salts were dissolved in ethanol (99%) to form a 1 M solution respectively, then a suitable amount of urea (98%) was added into the mixed metal solution to form a gel-like precursor (MU). In order to incorporate Fe, Fe solution mixed with urea was added in to NiMo precursor. Finally, a certain amount (5 wt.%) of Black Pearls 2000^®^ (BP) was added as additional carbon source into the metal precursor. The precursor mixture was then transferred into a crucible and heat treated in a furnace up to 800°C under a N_2_ flow (99.9%). After cooling down, a dark powder was obtained, identified as NiMoC@C or NiFeMo_2_C@C nanoparticles. Experimental details are shown in Supplementary Table S1.

### 2.2 Physical and chemical characterization

XRD data was collected on a PANalytical CubiX3 X-ray powder diffractometer. The theta-2theta scan measurements were carried out in standard reflection mode, using Cu K alpha radiation, with samples holders spinning on the stage during the scan. Match software was used to analyse the crystallographic phases, and ICDD standard database used as reference. XPS measurements were performed on ThermoFisher Scientific Nexsa XPS. It has a monochromated, micro-focused, low-power Al K-Alpha X-ray source. Raman spectroscopy was performed using a Renishaw in Via instrument, equipped with a 660 nm laser. The spectra were obtained by performing 4 acquisitions with 30 s of exposure. The parameters of high-resolution spectrum for C 1s, Ni 2p, Mo3d, Fe 2p: 50 eV pass energy, 30 scans, 50 s dwell time. Scanning electron microscopy (SEM) was performed on a FEI inspect F instrument. Transmission electron microscopy (TEM) images were taken using a Jeol’s JEM-F200 cold-FEG S/TEM with an operation voltage 200 kV.

### 2.3 Electrochemical test

The catalyst ink was prepared by grinding 2.5 mg of the powder catalyst and then adding 700 μl DIW, 280 ul ethanol (5 wt.% in lower aliphatic alcohols and 15%–20% water, Sigma Aldrich), 20 μl of Nafion^®^ solution. The ink was ultrasonicated for 1 h and then drop-cast 5 μl on glassy carbon (3 mm diameter) electrode and allowed to dry at room temperature for 30 min in ambient air. OER measurements were conducted using a three-electrode cell set-up. The Autolab potentiostat was employed with an Ag/AgCl (3 M KCl) as a reference electrode and platinum sheet as counter electrode. All the measurements were performed in O_2_-saturated 0.5 M KOH electrolyte at room temperature. Cyclic voltammetry (CV) measurements were performed in the range of −0.2–0.8 V (vs. Ag/AgCl) with a scan rate of 5 mV/s. Linear sweep voltammetry (LSV) were performed in the range of 0–1 V (vs. Ag/AgCl) with a scan rate of 5 mV/s. The polarization curves were iR corrected manually. Impedance measurements were performed in the frequency range from 100K Hz to 0.1 Hz. Stability measurement was conducted by chronopotentiometry at an applied current density of 10 mA/cm^2^. The potentials were converted to reversible hydrogen electrode (RHE) scale by using the following formula: E_RHE_ = E_0Ag/AgCl_ + E_Ag/AgCl_ + (0.059 × pH). The loading of catalysts was 0.177 mg/cm^2^ without adding any extra conductive reagent in the ink.

## 3 Results and discussion

**FIGURE 2:**
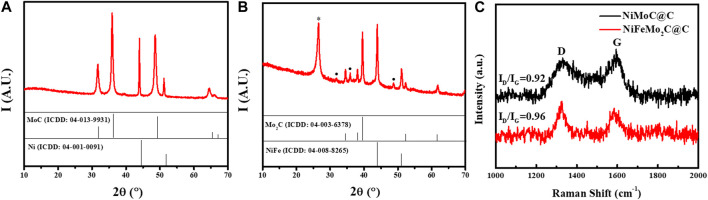
XRD patterns of as-prepared NiMo- **(A)** and NiFeMo- **(B)** based samples. **(C)** Raman spectra of the NiMoC@C and NiFeMo_2_C@C. The reference patterns of MoC (ICDD: 04–013–9931), NiFe (ICDD: 04–008–8265), Mo_2_C (ICDD: 04–003–6378), Ni (ICDD: 04–001–0091) are reported as vertical full lines, for comparison. The peak marked with * and • are attributed to carbon (ICDD: 04-15-2407), and MoC, respectively.

The as-prepared NiMoC@C and NiFeMo_2_C@C samples were characterised by X-ray powder diffraction (XRD) to give information on crystallinity and phase composition. Figures 2A,B report the XRD patterns of the binary and ternary systems. The reference patterns (from the ICDD database) are also reported for comparison (full vertical lines).

From the pattern of the NiMo-based sample (Figure 2A), it can be observed that the bimetallic system is made of two phases, which were indexed as MoC (peaks at 31.7^o^, 35.9^o^ and 48.6^o^, ICDD: 04–013–9931) and Ni° (peaks at 43.9^o^ and 51.2^o^, ICDD: 04–001–0091). In both phases, the observed peaks are slightly shifted (to a lower angle), compared to those expected from the database, which indicated: 1) lattice expansion, in both cases, and 2) indirectly, an interaction between the two systems. The average crystallite sizes of Ni and MoC, estimated by the Scherrer equation, were approximately 64 nm and 28 nm, respectively. Surprisingly, no carbon phase is observed, although its presence was confirmed by HRTEM and Raman analysis (*vide infra*).

Interestingly, the incorporation of Fe, to form the ternary systems seems to have a drastic effect on the final composition. As shown in Figure 2B, the MoC phase is now hardly observed (see • marked peaks) for it is replaced by the corresponding semi-carbide (Mo_2_C, peaks at 34.5^o^, 38.1^o^, 39.5^o^, 52.3^o^, ICDD: 04–003–6378). In addition, the peak of graphitic carbon is observed (26°, ICDD 04-015-2407), possibly due to the catalytic effect of iron on the graphitisation of carbon (Chen C and Wang, 2018; Destyorini et al., 2021). For comparison, the XRD patterns of the monometallic and bimetallic systems are reported in Supplementary Figure S1. The XRD patterns of the pure Ni-, the pure Mo-and the pure Fe-based samples show the formation of pure Ni° (Supplementary Figure S1A), Mo_2_C (Supplementary Figure S1B) and Fe_3_C (Supplementary Figure S1C) phases, respectively, as expected when the urea-glass-route was used (Giordano et al., 2008; Giordano et al., 2010; Clavel et al., 2014). The pattern of NiFe-sample (Supplementary Figure S1D) shows the formation of a NiFe alloy (peaks at 43.9° and 51.1°, ICDD 04–008–8265). Although the pattern of pure Ni and NiFe alloy are very similar, we can reasonably attribute the pattern to NiFe (rather than Ni°), as no other peaks attributable to a Fe phase are observed (iron must be then in combination with nickel).

As expected, a carbon phase (peak at ∼26°) is only observed alongside the pure Ni- and Fe_3_C phases. Interestingly, the pure Mo-based sample is always leading to a pure Mo_2_C phase (peaks at 43.8° and 50.9°), and the formation of pure MoC was never observed in absence of Ni, indirectly confirming the interaction between the two metals in the bimetallic system. Raman spectroscopy was employed to investigate the graphitization degree (G-band) and structural defects (D-band) of catalysts (Figure 2C), as discussed later on.

**FIGURE 3:**
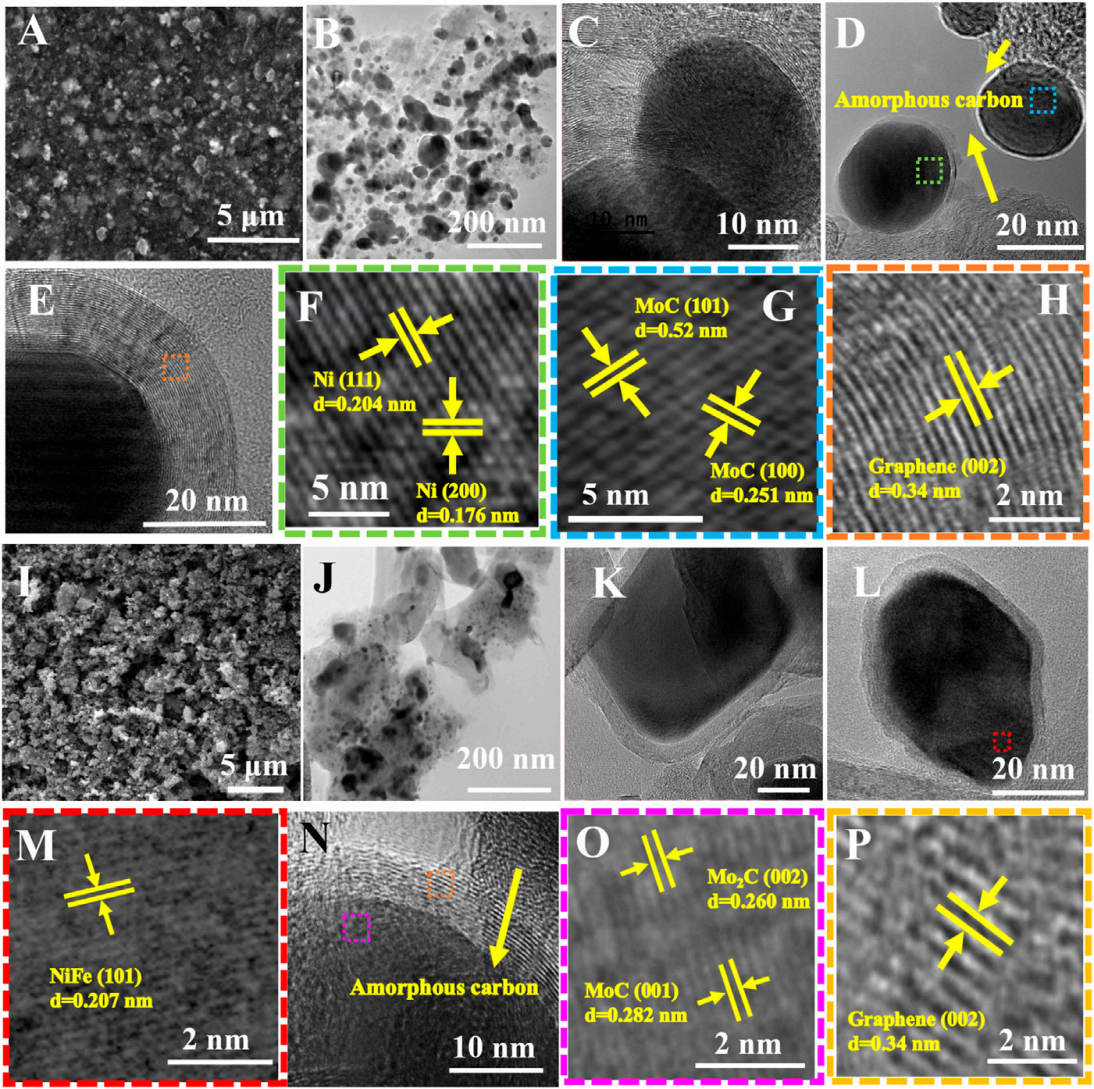
EM images of NiMoC@C **(A–H)** and NiFeMo_2_C@C **(I–P)** samples. **(A, I)** SEM images **(B, J)** low resolution TEM images. **(C–H)** HRTEM images of core-shell NiMoC@C samples. **(F–G)** HRTEM images of selected area in Figure 3D. **(F)** from area highlighted in green in Figure 3D. **(G)** from area highlighted in blue in Figure 3D. **(H)** HRTEM image of highlighted area (in orange) from Figure 3E. **(K–P)** HRTEM images of core-shell NiFeMo_2_C@C. **(M)** HRTEM image of selected area in Figure 3L, from area highlighted in red. **(O)** HRTEM image of selected area in Figure 3N: from area highlighted in pink. **(P)** HRTEM image of selected area in Figure 3N: from area highlighted in orange.

The morphological properties of NiMoC@C and NiFeMo_2_C@C systems were characterized by scanning electron microscopy (SEM) and transmission electron microscopy (TEM). Figure 3 reports the EM images of NiMoC@C (Figures 3A–H) and NiFeMo_2_C@C (Figures 3I–P) samples. SEM images give a general overview of the systems’ homogeneity, while TEM images allow a closer look, showing diverse particles (darker contrast) loaded onto a “lighter” matrix (reasonably made of amorphous carbon). The darker spots, expected to be the metallic phases (as confirmed by the elemental mapping analysis, *vide infra*) are relatively homogenous in shape (spheroidal). The lattice fringes can nicely be observed on HR-TEM images. The d-spacing (from the observed lattice fringes) of the NiMoC@C sample confirm the XRD outcomes, showing the presence of Ni and MoC planes, (111) and (200) for Ni (0.204 and 0.176 nm) and (101) and (100) (0.52 and 0.251 nm) for MoC phase. It is worth noting that the interface between Ni and MoC could not be unambiguously identified, possibly due to an overlapping of the two phase lattices. Interestingly, while the carbon around the metallic particles is visibly crystalline (Figures 3H,P) and identified as graphite (lattice plane distance of 0.34 nm, very close to the expected value for pure graphite (0.335 nm), the rest of the carbon is clearly amorphous (no lattice fringes observed).

TEM images of the ternary system (Figure 3J) show a less homogeneous system, both in size and shape, compared to the bimetallic one. This could be explained considering a disruptive effect of the incorporation of Fe into the NiMoC structure. Yet, also in this case, core-shell structure can be observed (Figures 3K,L,N). The d-spacing for the NiFeMo-based system (Figures 3M,O,P) confirmed the presence of the NiFe phase (0.207 nm) corresponding to the (101) facets of NiFe (Figure 3M), and the lattice distance of 0.260 nm and 0.282 nm corresponding to the (002) and (001) facets of Mo_2_C and MoC, respectively (Figure 3O).

**FIGURE 4:**
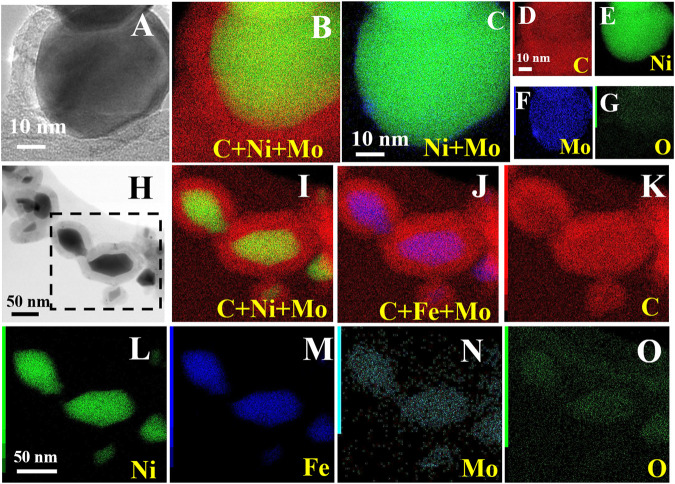
HRTEM image/bright field TEM images and the corresponding elemental mapping of C, Ni, Mo and O for NiMoC@C **(A–G)** and C, Ni, Fe, Mo and O for NiFeMo_2_C@C **(H–O)**.

To gain a further inside into the composition of the systems, elemental mapping images were recorded during TEM analysis (reported in Figure 4), for both NiMoC@C and NiFeMo_2_C@C. This study confirms the metallic nature of the core and the graphitic carbon shell. For both bi- and tri-metallic systems, the core is composed of Ni and Mo, or Ni, Fe and Mo elements, uniformly distributed and in close contact within the core, with the carbide phase being at the interface with the shell (Figure 4N shows a broader distribution of Mo, partially reaching the outer shell). It has been confirmed that the exposed metallic active sites in the shell can directly interact with the graphitic carbon shell (Ni et al., 2017), which could enhance the OER activity.

To have a better insight of the carbon phases, Raman was performed (Figure 2C). Information on graphitization degree and structural defects can be achieved *via* the so-called G-band and D-band (at ∼1,330 cm^-1^ and ∼1,590 cm^-1^, respectively), and by the ratio of their integrated area (I_D_/I_G_). From Figure 2C, it can be observed that the Raman carbon bands in the NiFeMo_2_C@C are more defined and sharper than in the NiMoC@C. Furthermore, the calculated I_D_/I_G_ is larger for the ternary system, than the bimetallic one (0.96 vs. 0.92, respectively). These findings indicate that in the ternary system the carbon phase is more defined than in the bimetallic one (which explains why no carbon peak was observed in the XRD pattern of NiMoC@C), but possibly turbostratic, i.e., less crystalline than pure graphite.

**FIGURE 5:**
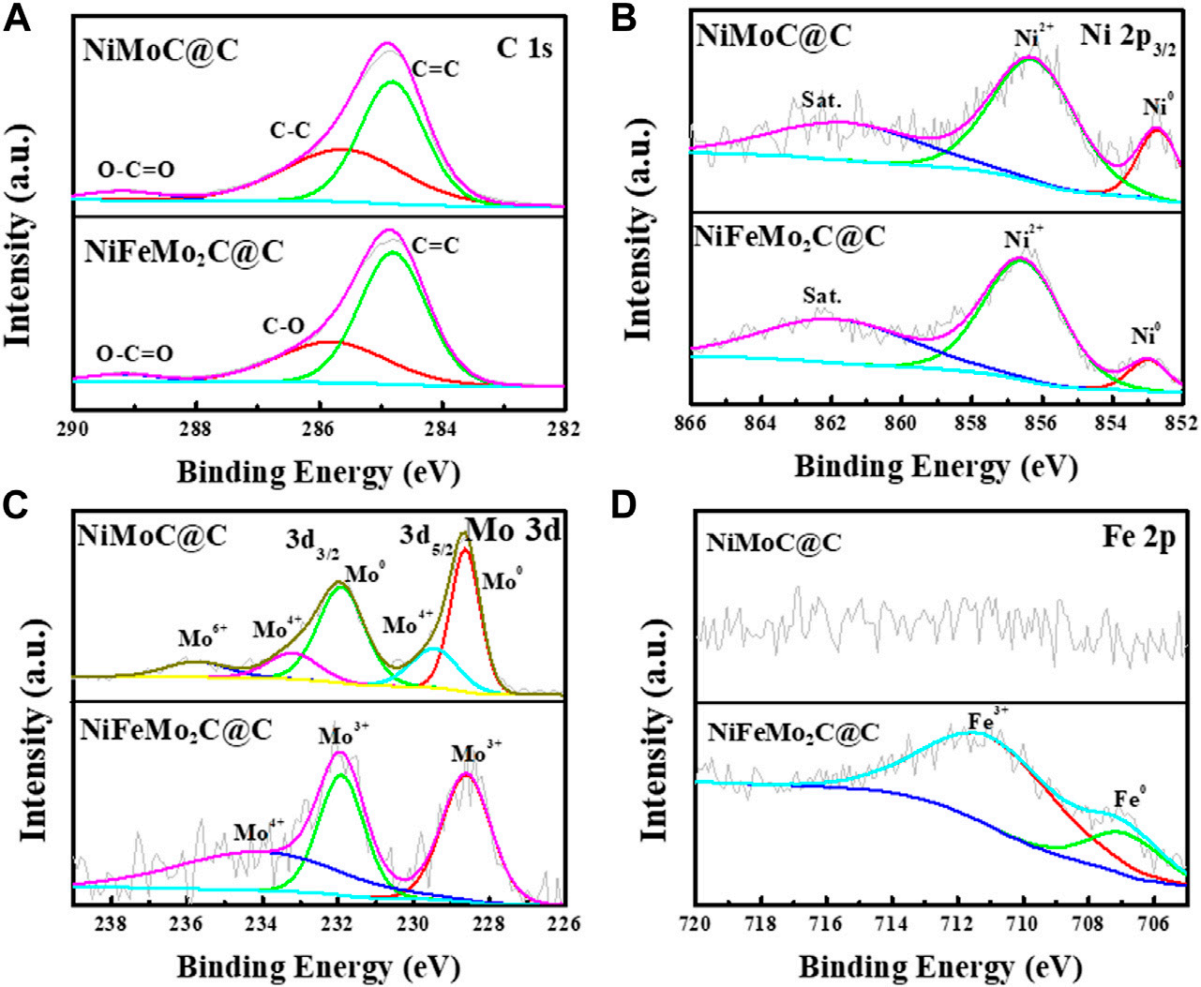
**(A)** High-resolution XPS spectra of C in NiMoC@C and NiFeMo_2_C@C, **(B)** Ni2p in NiMoC@C and NiFeMo_2_C@C, **(C)**High-resolution XPS spectra of Mo3d, **(D)** High-resolution XPS spectra of Fe2p.

The modulation of the electronic structures of the NiMoC@C and NiFeMo_2_C@C nanoparticles were determined by XPS analysis. In Figure 5A, the peaks are shown at around 284.8, 285.58, and 289.2 eV in NiMoC@C, 284.8, 285.8 and 289.2 eV in NiFeMo_2_C@C. The peak located at 284.8 eV is assigned to the sp^2^ C=C band of graphitized carbon, while the peaks at around 285.58, 285.8, and 289.2 eV are attributed to the C-C high (C–C sp^3^ bonds in the amorphous phase), C-O and COO bands, respectively (Smith et al., 2016; Destyorini et al., 2021). High-resolution Ni 2p spectra shows peaks of Ni2p_3/2_ at 852.66, 856.2 eV and shakeup satellites at 861.5 eV in NiMoC@C, while peaks of Ni2p_3/2_ at 852.97, 856.54 eV and shakeup satellites at 861.86 eV in NiFeMo_2_C@C (Figure 5B), which indicates that the chemical states of Ni is Ni^0^ (Biesinger et al., 2011; Geng et al., 2021) and Ni^2+^ of Ni(OH)_2_ (Bancroft, 2000) in both NiMoC@C and NiFeMo_2_C@C. High-resolution Mo3d spectra can be deconvoluted into five peaks in NiMoC@C, locating at 228.5 and 229.4 ev for Mo3d_5/2_, 231.86, 233.14 and 235.67 eV for Mo3d_3/2_, respectively (Figure 5C), which are assigned to Mo^0^, Mo^4+^ (MoO_2_), Mo^0^, Mo^4+^ (MoO_2_), Mo^6+^ (MoO_3_) (Pu et al., 2020; Zhang L. et al., 2021). The existence of Mo^0^ demonstrating the presence of Mo-Mo and Mo-C states of γ-MoC in NiMoC@C (Liu et al., 2023), in this case, the existence of a dominant Mo^0^ peak along with peaks of oxidation states of Mo (MoO_x_) can be explained that part of Mo on the surface were oxidized due to exposed to the air (Wan et al., 2014). Three peaks are shown at 228.6 eV (Mo^3+^), 231.91 eV (Mo^3+^), ascribing to β-Mo_2_C and 234.05 eV (Mo^4+^, MoO_2_) in NiFeMo_2_C@C (Wan et al., 2014; Li et al., 2022). This oxidation is inevitable for nanoscale materials because of the surface oxidation of MoC and Mo_2_C during operations. Furthermore, the shift of Ni2p and Mo3d peaks in NiFeMo_2_C@C is observed after Fe incorporation, spectrum of Ni in NiFeMo_2_C@C displays a visible positive shift of nearly 0.3 eV and 0.1 eV compared to the NiMoC@C, indicating the existence of strong electronic interactions between Ni and Fe species while slightly weak interactions exist between Mo and Fe, suggesting electrons transfer from Fe nanoparticles to Ni-Mo in NiFeMo2C@C, and the electron structure can be optimized by the incorporation of Fe. As expected and showed in Figure 5D, no Fe2p signal is observed in the NiMoC@C, while Fe 2p is observed in NiFeMo_2_C@C. The peaks at 706.85 and 710.98 eV in the Fe region belong to Fe^0^ and Fe^3+^ of Fe_2_O_3_ (Biesinger et al., 2011). The existence of Fe signal confirms the incorporation of Fe in NiFeMo_2_C@C. In addition, characteristic peaks of Ni^0^ and Fe^0^ were attributed to the presence of NiFe alloy in the catalyst structure. The element contents (wt.%) of different samples are shown in Supplementary Table S2. More amorphous carbon (sp^3^ C-C bond) was transformed into ordered graphitic carbon in NiFeMo_2_C@C (63.54 wt.%) than in NiMoC@C (53.33 wt.%), which leads to more amorphous carbon phase remains in NiMoC@C structure (42.11 wt.%) than in NiFeMo_2_C@C (32.33 wt.%), which is consistent with Raman results.

**FIGURE 6:**
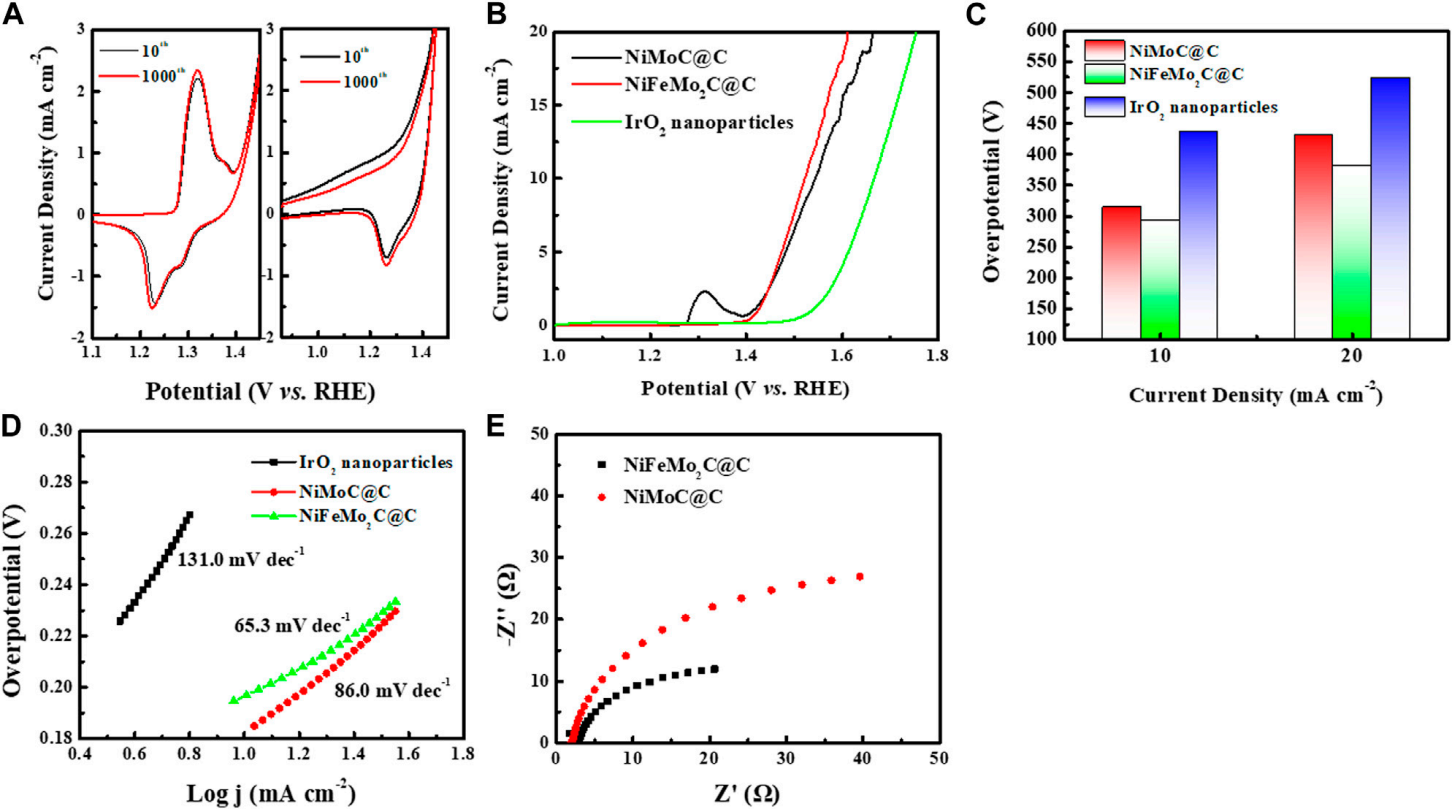
Electrochemical performance of as-prepared NiMoC@C, NiFeMo_2_C@C catalysts and commercial IrO_2_ NPs toward the OER in 0.5 M KOH solution. **(A)** CV curves of 10th and 1000th cycles of NiMoC@C, NiFeMo_2_C@C at a scan rate of 5 mV/s. **(B)** iR-compensated LSV curves of NiMoC@C, NiFeMo_2_C@C catalysts and commercial IrO_2_ NPs at a scan rate of 5 mV/s. **(C)** Summary of overpotential at j = 10 mA cm^-2^ and 20 mA cm^-2^. **(D)** Tafel plots determined from the LSV curves. **(E)** EIS measurements at a constant potential of 1.544 V and 1.522 V vs. RHE for NiMoC@C, NiFeMo_2_C@C respectively.

The OER performances of NiMoC@C and NiFeMo_2_C@C were investigated in a typical three-electrode system in O_2_-saturated 0.5 M KOH. Commercial IrO_2_ nanoparticles were also tested for comparison. The OER performance of monometallic Ni, Mo_2_C and Fe_3_C, and bimetallic NiFe are reported in Supplementary Figure S3 for comparison. The Cyclic Voltammograms (CV) corresponding to the 10th and 1000th cycles are shown in Figure 6A. The absence of significant differences between 10th cycle and 1000th cycles indicated a good durability.

Figure 6B shows the set of iR-corrected LSV curves recorded for various electrocatalysts at a scan rate of 5 mV S^−1^. NiFeMo_2_C@C catalysts require the lowest onset potential (1.522 V) to drive the forward or reverse reaction among them, which suggests a superior OER activity. NiMoC@C bimetallic nanoparticles have a higher requirement of onset potential (1.544 V). Overpotential and Tafel slope were calculated based on the iR-corrected LSV. From Figure 6C, NiFeMo_2_C@C delivers the lowest overpotential of 292 mV while the overpotential of NiMoC@C exhibits 314 mV at 10 mA cm^-2^. At current density of 20 mA cm^-2^, both catalysts present a relatively high overpotential. Both NiMoC@C and NiFeMo_2_C@C show a much better OER activity than commercial IrO_2_ nanoparticles, demonstrating their potential to replace the noble metal-based electrocatalysts. The NiFeMo_2_C@C affords a lower Tafel slope of 65.3 mV dec^−1^ compare to NiMoC@C (86.0 mV dec^−1^) in Figure 6D. The OER performance of monometallic Ni, Mo_2_C and Fe_3_C, and bimetallic NiFe alloy are reported in Supplementary Figure S3. The bimetallic NiFe catalysts shown the best performance (overpotential 355 mV@10 mA cm^-2^) towards the OER, and Ni shows a 385 mV of overpotential at 10 mA cm^-2^, while Mo and Fe carbides are not very active for the OER. The Tafel slope of NiFe (105.6 mV dec^−1^) is lower than Ni (122.0 mV dec^−1^) and the monometallic systems, which proves again the OER activity can be optimized with the incorporation of Fe. Electrochemical impedance spectroscopy (EIS) was performed to investigate the OER kinetics, shown in Figure 6E. The charge-transfer resistances (R_ct_) of NiFeMo_2_C@C reaches the small value of 32.6 Ω at an overpotential of 292 mV vs. RHE. Both small Tafel slope and R_ct_ value indicating that NiFeMo_2_C@C has fast reaction kinetics, which supports enhanced OER activity.

The NiFeMo_2_C@C catalyst shown a better OER activity, which can be attributed to Fe incorporation. When Fe is incorporated into the NiMoC lattice, the electronic structure is optimised, electrons can be transfered from Fe nanoparticles to Ni-Mo in NiFeMo_2_C@C that improves the intrinsic OER activity (Trotochaud et al., 2014; Corrigan, 2019). The Ni or Mo in NiFeMo_2_C@C appear to have stronger oxidizing power than that in NiMoC@C, resulting in faster OER kinetics and better OER activity (Trotochaud et al., 2014). Additionally, NiFe catalyst has been proven theoretically to be optimal for the OER (Diaz-Morales et al., 2015; Gorlin et al., 2016). Supplementary Table S3 shows a comparison of NiMo-based catalysts tested in alkaline electrolyte toward the OER reported in the literature and compared to the present study.

**FIGURE 7:**
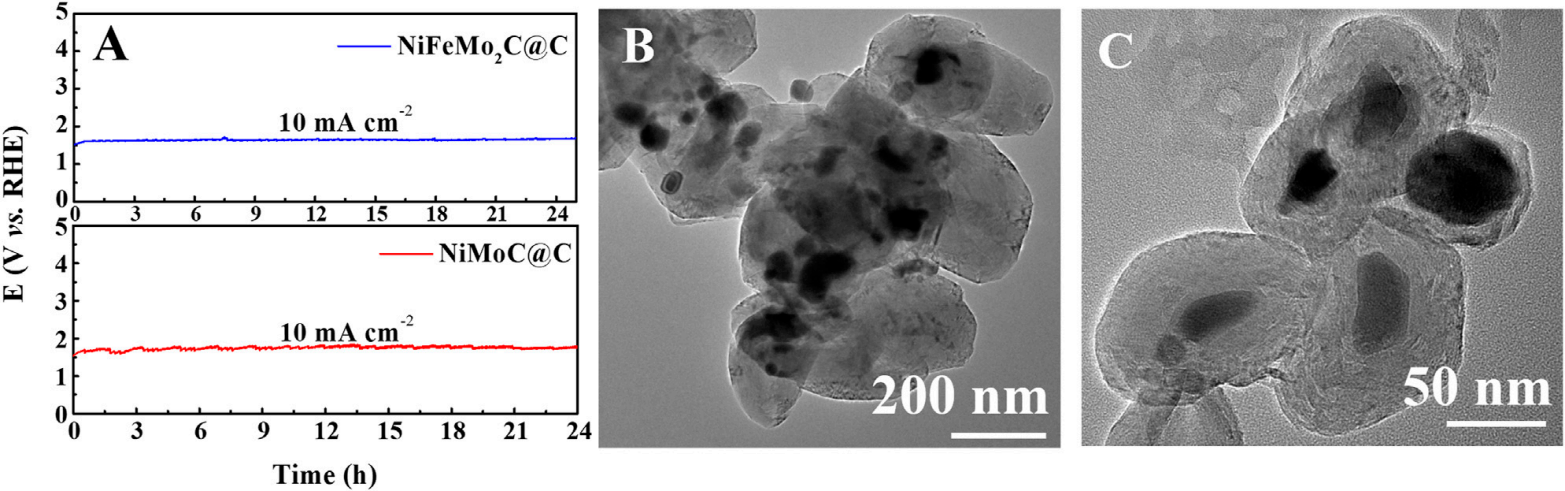
The chronopotentiometry tests at 10 mA cm-2 in 0.5 M KOH for 24 h **(A)**. TEM images of NiFeMo2C@C after OER process **(B, C)**.

The long-term stability of the NiMoC@C and NiFeMo_2_C@C in 0.5 M KOH electrolyte was tested *via* chronopotentiometry (Figure 7A). The potential of 1.522 V and 1.544 V were given to reach a current density at 10 mA cm^-2^ respectively. The potential shows no significant changes after working for at least 24 h, which proves the catalytic durability of the samples. TEM images of NiFeMo_2_C@C taken after 24 h of the OER process are shown in Figures 7B,C. These images show that the core-shell structure is preserved and nanoparticles do not aggregate, possibly due to the presence of the shell, Nanoscaled catalytic materials in fact tend to undergo aggregation during durability tests, which leads to the reduction of their electrocatalytic performance (Ouyang et al., 2018).

## 4 Conclusion

In this work, a facile pathway to prepare multi-metallic based systems as OER catalysts has been presented. The systems are found to have comparable properties to noble-metal catalysts and exhibit excellent stability. In particular, the prepared ternary transition metal electrocatalysts with core-shell structure are reported for the first time for the OER process. Both systems, NiMoC@C and NiFeMo_2_C@C, possess an unique core-shell structure. The obtained NiFeMo_2_C@C was shown to be stable and active for the OER under alkaline conditions, while the incorporation of Fe showed a positive effect in terms of activity, resulting in an improved OER activity that delivers an overpotential of 292 mV with a Tafel slope of 65.3 mV dec^−1^ at a current density of 10 mA cm^-2^ in 0.5 M KOH electrolyte. The improved OER performance of NiFeMo_2_C@C was attributed to the synergistic effect between NiFe and Mo carbide. In addition, the graphitic shell provides additional mechanical strength and chemical stability under alkaline OER conditions, at the same time, the rich defects structure of ternary alloy can effectively promote the charge transfer and mass transfer from NiFeMo_2_C@C. Concurrently, the NiMoC@C and NiFeMo_2_C@C remains stable during OER process in alkaline electrolyte for at least 24 h, making them promising candidates as electrocatalysts for OER process. This study helps to expand the application of the polymetallic alloy and core-shell structure nanomaterials in energy conversion, and it also provides an effective way for the electronic structures design of non-noble metals encapsulated in graphene layers. This work also suggests that, in the future, by optimizing the encapsulated ternary metal proportion and composition, more interface in the bi/ternary core and more electroactive sites in the shell may be achieved.

## Data Availability

The original contributions presented in the study are included in the article/Supplementary Material, further inquiries can be directed to the corresponding author.
