# Supplementary material for: Designed NiMoC@C and NiFeMo2C@C core-shell nanoparticles for oxygen evolution in alkaline media
Source: Front Chem. 2023 Apr 26;11:1162675. doi: 10.3389/fchem.2023.1162675 (PMC10169681; doi:10.3389/fchem.2023.1162675)
Supplement: Supplementary file 1 [file DataSheet1.pdf]

Supporting Information

TABLE S1

Experimental details of catalysts prepared by urea-glass-route.

Precursor	Metal Molar ratio	Urea/Metal molar ratio	Main phases from XRD
NiMoUrea@C	1:1	7	MoC, Ni
NiFeMoUrea@C	1:1:1	7	Mo_2C, NiFe, C, MoC

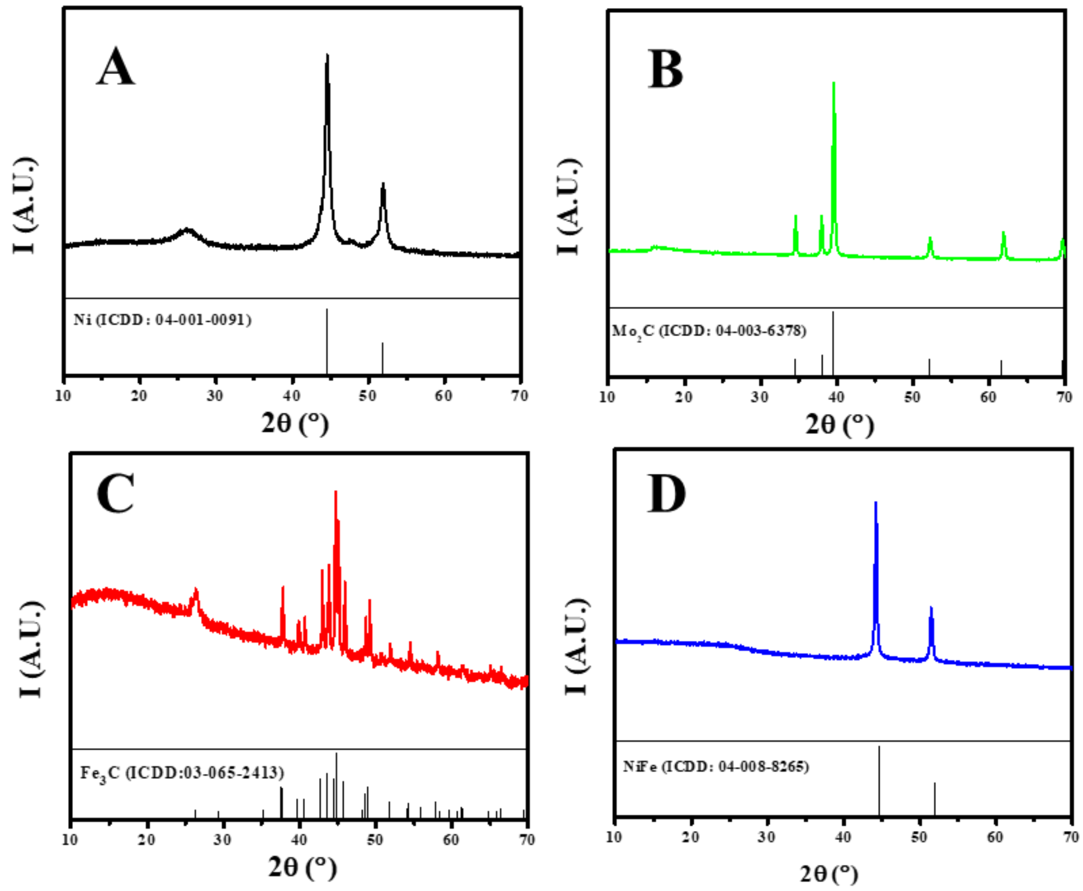

FIGURE S1

XRD pattern of **(A)** Pure Ni (from Ni-Urea@C precursor), **(B)** Mo_2C sample (from Mo-Urea@C precursor), **(C)** Fe_3C (from Fe-Urea@C precursor), **(D)** NiFe (from NiFe-Urea@C precursor). The corresponding reference patterns from ICDD are included for comparison.

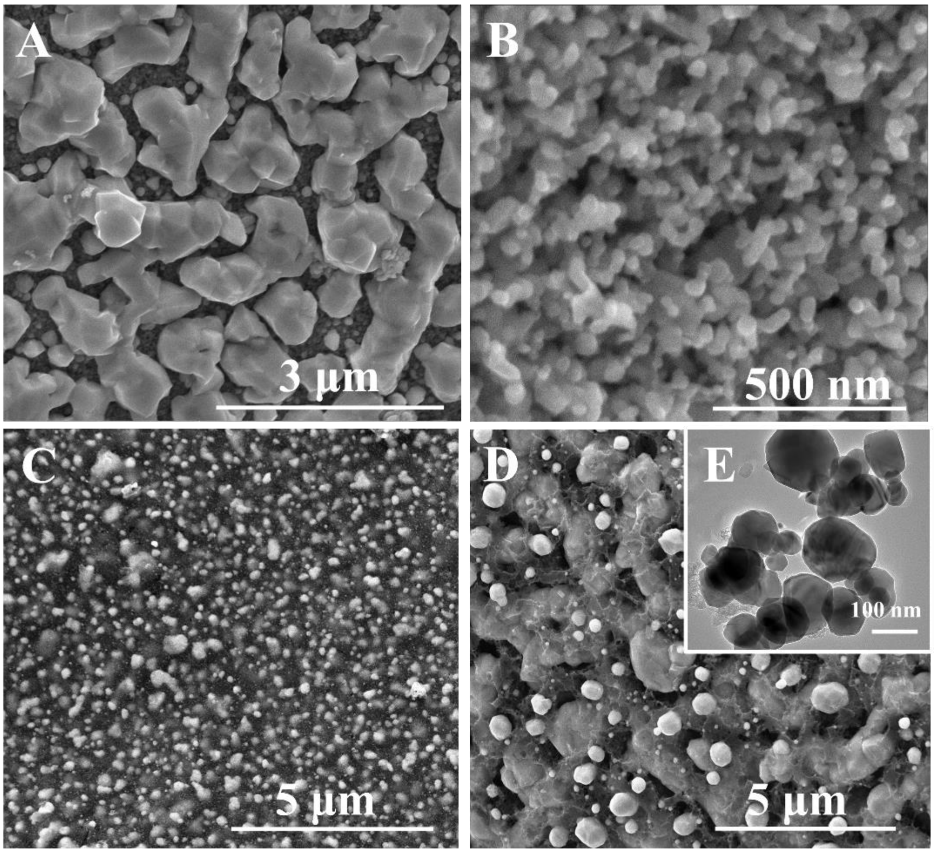

FIGURE S2

SEM images of samples identified (from XRD) as **(A)** metallic Ni, **(B)** Mo_2C, **(C)** Fe_3C, **(D)** NiFe and **(E)** TEM image of NiFe.

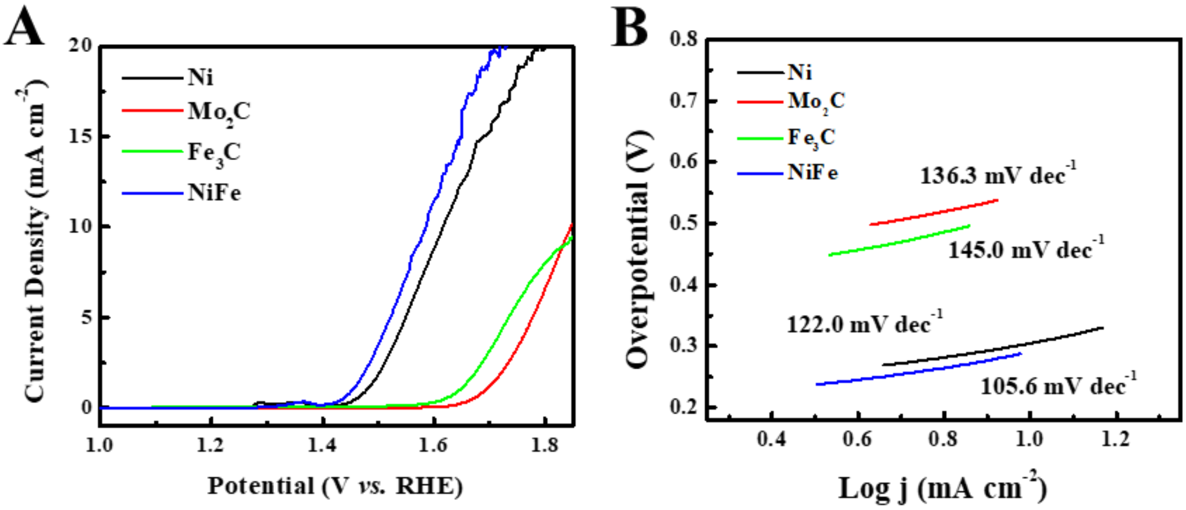

FIGURE S3

Electrochemical performance of monometallic Ni, Mo_2C and Fe_3C and bimetallic NiFe in 0.5 M KOH solution. **(A)** iR-compensated Linear sweep voltammetry (LSV) curves at a scan rate of 5 mV/s. **(B)** Tafel plots determined from the LSV curves.

TABLE S2

Element contents of different samples analyzed by XPS and binding energies obtained from XPS compared with literature

Elements	Attributions	NiMoC@C		$NiFeMo_2C$@C		Ref
		Contents (wt.%)	Peak positions (eV)	Contents (wt.%)	Peak positions (eV)	
C	C=C	53.33	284.8	63.54	284.8	(Smith et al., 2016; Destyorini et al., 2021)
	C-C/C-O	42.11	285.58	32.33	285.8	
	COO	4.56	289.2	4.13	289.2	
Ni	Ni^0	15	852.66	7.12	852.97	(Bancroft, 2000; Biesinger et al., 2011)
	Ni^{2+}	54.98	856.2	54.84	856.54	
	Sat.	30.03	861.5	38.04	861.86	
Mo	Mo^0	37.90	228.5, 231.86	-	-	(Wan et al., 2014; Pu et al., 2020; Li et al., 2022)
	Mo^{3+}	-	-	65.14	228.6, 231.91	
	Mo^{4+}	45.4	229.4, 233.14	34.86	234.05	
	Mo^{6+}	7.52	235.67	-	-	
Fe	Fe^0	-	-	21	706.85	(Biesinger et al., 2011)
	Fe^{3+}	-	-	79	710.98	

TABLE S3

The performance of NiMo-based bimetallic/trimetallic electrocatalysts for OER.

Electrocatalysts	Electrolyte	Overpotential@10 mA cm^{-2}	Tafel slope (mV dec^{-1})	Ref
NiMoC@C	0.5 M KOH	314	86.0	This work
$NiFeMo_2C$@C	0.5 M KOH	292	65.3	This work
NiMoO4@$MoSe_2$/Ni_xSe_y-6/NF*	1 M KOH	290 (200 mA cm^{-2})	-	(Zhang et al., 2020)
NF@NiMoCo nanowire	1.0 M KOH	277	87	(Hu et al., 2019)
Mo-Ni_3S_2/Ni_xP_y/NF	1 M KOH	238 (50 mA cm^{-2})	60.6	(Luo et al., 2020)
Mo–Ni/NC	1 M KOH	270	64.3	(Zhao et al., 2022)
Ni/Mo-Ni microrods	1 M KOH	215	39	(Li et al., 2022)
NiMo-FG	1.0 M KOH	338	67	(Jeong et al., 2019)
O-MoNi-C/NF	1 M KOH	190	65.35	(Zu et al., 2019)
NiFeMo IOS*@NF	1 M KOH	198	36	(Hsieh et al., 2020)
MOF-derived ZIF-67/NiMoCo/CNT	1.0 M KOH	306	70	(Zhang C, Available at SSRN 4049709.)

NiMoFe<br>NiMoFeP	1.0 M KOH	337<br>286	57<br>28	(Baek et al., 2019)
NiMoFe/Cu nanowire	1.0 M PBS	520	148.53	(Cao et al., 2022)
NiFeMo oxides	0.1 M KOH	280	49	(Duan et al., 2019)
Ni/NiFeMoO$_x$/NF	1 M KOH	255	35	(Li et al., 2020)
NiMo-Fe	1.0 M KOH	217	30.05	(Liao et al., 2022)
NiFeMo alloy	1.0 M KOH	238	35	(Qin et al., 2018)
Ni-Fe-MoN Nanotubes	0.1 M KOH	228	41	(Zhu et al., 2018)
Ni-Mo-Fe	1 M KOH	344	45	(Badrnezhad et al., 2021)
Ni-Fe-W-Mo alloy	5.35 M KOH	152	-	(Zhang et al., 2021)

*NF: Nickel foam, IOS: inverse opal structure

References

Badrnezhad, R., Nasri, F., Pourfarzad, H., and Jafari, S.K. (2021). Effect of iron on Ni–Mo–Fe composite as a low-cost bifunctional electrocatalyst for overall water splitting. *International Journal of Hydrogen Energy* 46(5)**,** 3821-3832. doi: 10.1016/j.ijhydene.2020.10.174.

Baek, M., Kim, G.W., Park, T., and Yong, K. (2019). NiMoFe and NiMoFeP as Complementary Electrocatalysts for Efficient Overall Water Splitting and Their Application in PV-Electrolysis with STH 12.3. *Small* 15(49)**,** 1905501. doi: 10.1002/smll.201905501.

Bancroft, H.W.N.á.D.L.á.G.M. (2000). Interpretation of Ni2p XPS spectra of Ni conductors and Ni insulators. *Phys Chem Minerals* 27**,** 357-366. doi: 10.1007/s002690050265.

Biesinger, M.C., Payne, B.P., Grosvenor, A.P., Lau, L.W.M., Gerson, A.R., and Smart, R.S.C. (2011). Resolving surface chemical states in XPS analysis of first row transition metals, oxides and hydroxides: Cr, Mn, Fe, Co and Ni. *Applied Surface Science* 257(7)**,** 2717-2730. doi: 10.1016/j.apsusc.2010.10.051.

Cao, X., Fan, R., Zhou, J., Chen, C., Xu, S., Zou, S., et al. (2022). NiMoFe/Cu nanowire core-shell catalysts for high-performance overall water splitting in neutral electrolytes. *Chem Commun (Camb)* 58(10)**,** 1569-1572. doi: 10.1039/d1cc06409f.

Destyorini, F., Irmawati, Y., Hardiansyah, A., Widodo, H., Yahya, I.N.D., Indayaningsih, N., et al. (2021). Formation of nanostructured graphitic carbon from coconut waste via low-temperature catalytic graphitisation. *Engineering Science and Technology* 24(2)**,** 514-523. doi: 10.1016/j.jestch.2020.06.011.

Duan, Y., Yu, Z.Y., Hu, S.J., Zheng, X.S., Zhang, C.T., Ding, H.H., et al. (2019). Scaled-Up Synthesis of Amorphous NiFeMo Oxides and Their Rapid Surface Reconstruction for Superior Oxygen Evolution Catalysis. *Angew Chem Int Ed Engl* 58(44)**,** 15772-15777. doi: 10.1002/anie.201909939.

Hsieh, C.-T., Huang, C.-L., Chen, Y.-A., and Lu, S.-Y. (2020). NiFeMo alloy inverse-opals on Ni foam as outstanding bifunctional catalysts for electrolytic water splitting of ultra-low cell voltages at high current densities. *Applied Catalysis B: Environmental* 267**,** 118376. doi: 10.1016/j.apcatb.2019.118376.

Hu, K., Wu, M., Hinokuma, S., Ohto, T., Wakisaka, M., Fujita, J.-i., et al. (2019). Boosting electrochemical water splitting via ternary NiMoCo hybrid nanowire arrays. *Journal of Materials Chemistry A* 7(5)**,** 2156-2164. doi: 10.1039/c8ta11250a.

Jeong, S., Hu, K., Ohto, T., Nagata, Y., Masuda, H., Fujita, J.-i., et al. (2019). Effect of Graphene Encapsulation of NiMo Alloys on Oxygen Evolution Reaction. *ACS Catalysis* 10(1)**,** 792-799. doi: 10.1021/acscatal.9b04134.

Li, H., Cai, C., Wang, Q., Chen, S., Fu, J., Liu, B., et al. (2022). High-performance alkaline water splitting by Ni nanoparticle-decorated Mo-Ni microrods: Enhanced ion adsorption by the local electric field. *Chemical Engineering Journal* 435**,** 134860. doi: 10.1016/j.cej.2022.134860.

Li, Y.K., Zhang, G., Lu, W.T., and Cao, F.F. (2020). Amorphous Ni-Fe-Mo Suboxides Coupled with Ni Network as Porous Nanoplate Array on Nickel Foam: A Highly Efficient and Durable Bifunctional Electrode for Overall Water Splitting. *Adv Sci (Weinh)* 7(7)**,** 1902034. doi: 10.1002/advs.201902034.

Liao, H., Zhang, X., Niu, S., Tan, P., Chen, K., Liu, Y., et al. (2022). Dynamic dissolution and re-adsorption of molybdate ion in iron incorporated nickel-molybdenum oxyhydroxide for promoting oxygen evolution reaction. *Applied Catalysis B: Environmental* 307**,** 121150. doi: 10.1016/j.apcatb.2022.121150.

Luo, X., Ji, P., Wang, P., Cheng, R., Chen, D., Lin, C., et al. (2020). Interface Engineering of Hierarchical Branched Mo-Doped Ni_3S_2/Ni_xP_y Hollow Heterostructure Nanorods for Efficient Overall Water Splitting. *Advanced Energy Materials* 10(17)**,** 1903891. doi: 10.1002/aenm.201903891.

Pu, J., Cao, J., Ma, L., Zhou, K., Yu, Z., Yin, D., et al. (2020). Novel three-dimensional Mo2C/carbon nanotubes composites for hydrogen evolution reaction. *Materials Letters* 277**,** 128386. doi: 10.1016/j.matlet.2020.128386.

Qin, F., Zhao, Z., Alam, M.K., Ni, Y., Robles-Hernandez, F., Yu, L., et al. (2018). Trimetallic NiFeMo for Overall Electrochemical Water Splitting with a Low Cell Voltage. *ACS Energy Letters* 3(3)**,** 546-554. doi: 10.1021/acsenergylett.7b01335.

Smith, M., Scudiero, L., Espinal, J., McEwen, J.-S., and Garcia-Perez, M. (2016). Improving the deconvolution and interpretation of XPS spectra from chars by ab initio calculations. *Carbon* 110**,** 155-171. doi: 10.1016/j.carbon.2016.09.012.

Wan, C., Regmi, Y.N., and Leonard, B.M. (2014). Multiple phases of molybdenum carbide as electrocatalysts for the hydrogen evolution reaction. *Angew Chem Int Ed Engl* 126(25)**,** 6525-6528. doi: 10.1002/anie.201402998.

Zhang C, X.Z., Yu Y, et al. (Available at SSRN 4049709.). Mof-Derived Carbon Nanotubes Modified Nimoco Ternary Alloy Electrocatalyst for Efficient and Stable Overall Water Splitting. doi: 10.2139/ssrn.4049709.

Zhang, P., Tan, W., He, H., and Fu, Z. (2021). Binder-free quaternary Ni–Fe–W–Mo alloy as a highly efficient electrocatalyst for oxygen evolution reaction. *Journal of Alloys and Compounds* 853**,** 157265. doi: 10.1016/j.jallcom.2020.157265.

Zhang, Z., Ye, S., Ji, J., Li, Z., and Wang, F. (2020). Core/shell -structured NiMoO4 @ $MoSe_2/Ni_xSe_y$ Nanorod on Ni Foam as a Bifunctional Electrocatalyst for Efficient Overall Water Splitting. *Colloids and Surfaces A: Physicochemical and Engineering Aspects* 599**,** 124888. doi: 10.1016/j.colsurfa.2020.124888.

Zhao, M., Shen, X., Zhou, H., Wang, X., Wei, Z., Lv, J., et al. (2022). Bimetal Mo–Ni/NC as an effective electrocatalyst with accelerated kinetics for the oxygen evolution reaction. *Journal of Physics and Chemistry of Solids* 165**,** 110651. doi: 10.1016/j.jpcs.2022.110651.

Zhu, C., Yin, Z., Lai, W., Sun, Y., Liu, L., Zhang, X., et al. (2018). Fe-Ni-Mo Nitride Porous Nanotubes for Full Water Splitting and Zn-Air Batteries. *Advanced Energy Materials* 8(36)**,** 1802327. doi: 10.1002/aenm.201802327.

Zu, M.Y., Wang, C., Zhang, L., Zheng, L.R., and Yang, H.G. (2019). Reconstructing bimetallic carbide Mo6Ni6C for carbon interconnected MoNi alloys to boost oxygen evolution electrocatalysis. *Materials Horizons* 6(1)**,** 115-121. doi: 10.1039/c8mh00664d.
